# A Metabolic Landscape for Maintaining Retina Integrity and Function

**DOI:** 10.3389/fnmol.2021.656000

**Published:** 2021-04-15

**Authors:** Filipe O. Viegas, Stephan C. F. Neuhauss

**Affiliations:** ^1^Department of Molecular Life Sciences, University of Zurich, Zurich, Switzerland; ^2^Life Science Zurich Graduate School, Ph.D. Program in Molecular Life Sciences, Zurich, Switzerland

**Keywords:** metabolism, retina, brain, photoreceptor, glucose metabolism

## Abstract

Neurons have high metabolic demands that are almost exclusively met by glucose supplied from the bloodstream. Glucose is utilized in complex metabolic interactions between neurons and glia cells, described by the astrocyte-neuron lactate shuttle (ANLS) hypothesis. The neural retina faces similar energy demands to the rest of the brain, with additional high anabolic needs to support continuous renewal of photoreceptor outer segments. This demand is met by a fascinating variation of the ANLS in which photoreceptors are the central part of a metabolic landscape, using glucose and supplying surrounding cells with metabolic intermediates. In this review we summarize recent evidence on how neurons, in particular photoreceptors, meet their energy and biosynthetic requirements by comprising a metabolic landscape of interdependent cells.

## Introduction to Glucose Metabolism

Although cells fulfill a plethora of different functions, their functionality ultimately hinges upon the cell’s ability to generate energy for survival and cell division. The energy currency of cells is ATP, enabling thermodynamically unfavorable biological reactions. Depending on the availability of resources, unicellular organisms are able to shift their metabolism between consuming nutrients to produce energy (catabolism) and using this energy to produce the building blocks of a new cell (anabolism). Multicellular organisms experience similar demand-driven shifts in metabolism. These are particularly prominent in dividing tissue but also occur in postmitotic cells, which can shift toward anabolic states (Vander Heiden et al., 2009; Lunt and Vander Heiden, 2011).

**FIGURE 1:**
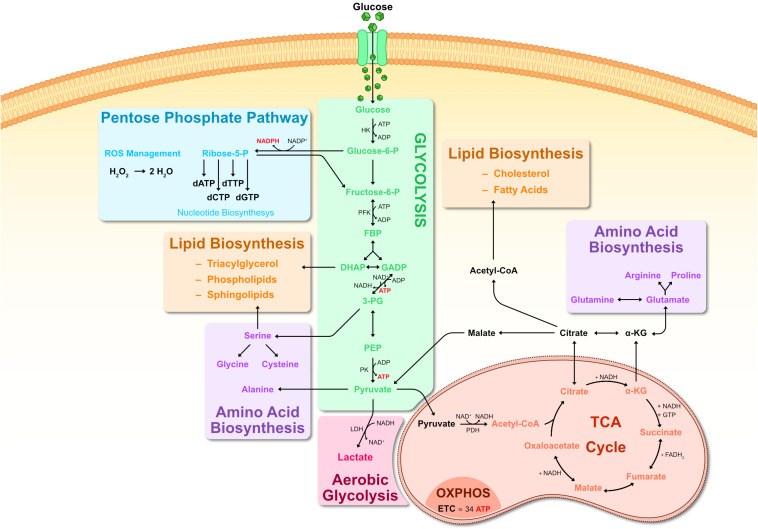
Metabolic pathways of glucose metabolism. Representation of key steps in glucose metabolism. Upon entering the cell, glucose is first metabolized by HK into G6P, consuming ATP. G6P can branch off into the PPP pathway, producing R5P and generating large amounts of NADPH as a byproduct. R5P is a precursor for creating nucleic acids. NADPH generated by the PPP can be used to scavenge ROS via glutathione reduction, converting H_2_O_2_ into H_2_O. G6P is further metabolized into F6P and subsequently into FBP. FBP is metabolized into one molecule of DHAP and another of GADP. DHAP can branch off into lipid biosynthesis where it is a precursor for phospholipids and triacylglycerol. Otherwise, DHAP is transformed into GADP and both GADP molecules are metabolized into 3-PG, generating ATP in the process. 3-PG can be used for serine biosynthesis, which can then contribute toward synthesis of sphingolipids as well as glycine and cysteine. 3-PG goes on to be metabolized into PEP and then into pyruvate by PK activity. Pyruvate can: serve as substrate for alanine biosynthesis; be converted to lactate by LDH (known as anaerobic respiration or aerobic glycolysis); enter a mitochondrion where PDH metabolizes it into Acetyl-CoA. Acetyl-CoA then enters the TCA cycle, where it is metabolized in a series of sequential reactions resulting in citrate, α-KG, succinate, fumarate, malate, oxaloacetate which cycles back to citrate. Some of these reactions generate byproducts such as NADH and FADH_2_. NADH and FADH_2_ are then used for OXPHOS, generating ATP in the inner mitochondrial membrane matrix. Abbreviations: α-KG, α-ketoglutarate; 3-PG, 3-phosphoglycerate; ADP, adenosine diphosphate; ATP, adenosine triphosphate; CoA, coenzyme A; dATP, deoxyadenosine triphosphate; dCTP, deoxycytidine triphosphate; dGTP, deoxyguanosine triphosphate; DHAP, dihydroxyacetone phosphate; dTTP, deoxythymidine triphosphate; ETC, electron transport chain; F6P, Fructose-6-phosphate; FADH, flavin adenine dinucleotide, reduced; FBP, fructose 1,6-biphosphate; G6P, glucose-6-phosphate; GADP, glyceraldehyde-3-phosphate; GTP, guanosine triphosphate; HK, hexokinase; LDH, lactate dehydrogenase; NAD, nicotinamide adenine dinucleotide; NADH, nicotinamide adenine dinucleotide, reduced; NADP, nicotinamide adenine dinucleotide phosphate; NADPH, nicotinamide adenine dinucleotide phosphate, reduced; OXPHOS, oxidative phosphorylation; PEP, phosphoenolpyruvate; PDH, pyruvate dehydrogenase; PK, pyruvate kinase; PPP, pentose phosphate pathway; TCA, tricarboxylic acid.

Catabolic reactions essentially consist of breaking-down molecules into smaller components that are that are oxidized to either provide energy or create building blocks for anabolic reactions. The most widely used carbon source is glucose, which is eventually catabolized to gain ATP, but can also provide crucial precursors for biosynthetic reactions. There are two major routes of glucose metabolism: Either, glucose feeds into glycolysis, where it is oxidized and becomes pyruvate, providing ATP and metabolic intermediates; or alternatively, glucose is shuttled from glycolysis into the pentose phosphate pathway for nucleic acid synthesis and the formation of NADPH (Figure 1).

Glycolysis is carried out by a set of cytoplasmic enzymes ultimately yielding pyruvate, catalyzing the phosphorylation of two ADP to ATP and causing the reduction of two NAD^+^ to NADH (Figure 1). In the presence of oxygen, pyruvate is oxidized to Acetyl-CoA and enters the mitochondrial tricarboxylic acid (TCA) cycle (also known as Krebs cycle or citric acid cycle). In the TCA cycle there are a series of oxidative reactions resulting in the generation of reductive power in the form of NADH and FADH_2_ (Figure 1). The electron transport chain in the membrane of the mitochondria then uses this reductive power to generate ATP in a process termed oxidative phosphorylation (OXPHOS). This is the most profitable component of energy metabolism since one glucose molecule generates up to 36 molecules of ATP (Figure 1).

When oxygen is limited, cells shift pyruvate away from the TCA and OXPHOS by reducing it to lactate in a process termed anaerobic respiration. Under oxygen-deprived conditions, NADH can no longer be reduced to NAD^+^. Since NAD^+^ is still required as an electron accepter, pyruvate is reduced to lactate, consuming NADH and replenishing the pool of NAD^+^ in the process. Under special circumstances, anaerobic respiration can also occur when oxygen is abundantly available, as will be detailed below.

In addition to being the backbone of energy metabolism, glycolysis and the TCA cycle also provide essential precursors for biosynthetic pathways. Several glycolytic intermediates contribute toward amino acid biosynthesis, nucleotide biosynthesis, complex carbohydrate biosynthesis, as well as lipid biosynthesis (Figure 1). The first step of glycolysis converts glucose into Glucose-6-phosphate (G6P) by hexokinase (HK) enzymes. G6P can either be ultimately metabolized to pyruvate, or be diverted to the pentose phosphate pathway (PPP), which provides the building blocks for nucleotides as well as replenishing NADPH (Figure 1). The other noteworthy biosynthetic contribution of glycolysis is generating precursors for phospholipid synthesis. The glycolytic intermediate dihydroxyacetone phosphate (DHAP) can be oxidized to glycerol-3-phosphate, which is an essential precursor of diacyl glycerophospholipids and glycerophospholipids, structural components of all biological membranes. Similarly to glycolysis, the TCA cycle provides important precursors for lipid and amino acid biosynthesis (Figure 1).

While the catabolic and anabolic roles of the described pathways are essential for all cells, variations serving different metabolic requirements exist. This is particularly true for neurons.

Due to their particular physiology and function, neurons are energetically demanding and rely almost exclusively on glucose for the energy they need (Ames, 2000; Dienel, 2019). The particular importance of neuronal energy metabolism stems from the steep energy requirements of neuronal signaling. Neurons function by transiently changing their membrane potential, which in turn poses a high metabolic burden on regaining resting potential through the activity of ATP-dependent ion pumps.

Next, we will focus on energy metabolism of neurons in the brain and the retina, and attempt to present a cohesive model of retinal energy metabolism.

## Brain Energy Metabolism

**FIGURE 2:**
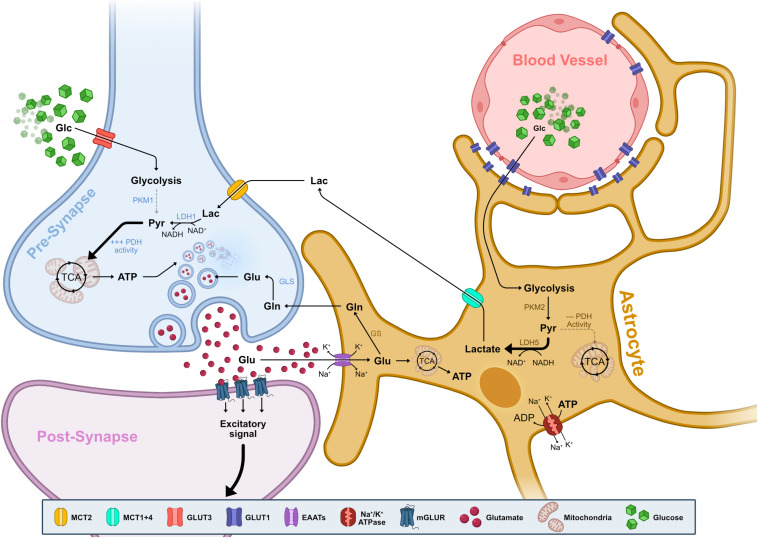
Neuron-Astrocyte metabolic interdependence. Representation of the metabolite flow between blood vessels, astrocytes and synaptic terminals of neurons. Glucose from the blood supply transverses the vessel endothelium and enveloping astrocyte endfeet via GLUT1. Glucose then enters aerobic glycolysis, with marginal contribution toward the TCA due to reduced PDH activity. Resulting pyruvate is almost exclusively transformed into lactate via the activity of LDH5. LDH5 is composed of LDHA subunits which preferentially drive the reaction toward consuming NADH and creating lactate, replenishing the NAD^+^ pool for increased glycolytic activity. Lactate is then exported via MCT1 and MCT4 and it is taken up by neurons via MCT2. In the neuron, lactate is metabolized back into pyruvate via LDH1. LDH1 is mainly composed of LDHB subunits which preferentially drive the reaction toward consuming NAD^+^ and creating pyruvate, replenishing the pool of available NADH. Pyruvate fuels the TCA cycle and OXPHOS, producing ATP necessary for replenishing pre-synaptic glutamate vesicles. Upon neuronal activation, glutamate is released into the synapse and binds to post-synaptic mGLUR GPCRs, ultimately resulting in a Ca^2+^ influx and signal propagation. Free glutamate in the synaptic cleft is re-uptaken by adjacent astrocytes via EAATs which co-transport Na^+^ and K^+^. Glutamate enters the glutamate-glutamine cycle and is metabolized into glutamine by GS. Glutamine is then exported and taken up by neurons, where it is turned back into glutamate by GLS and is used to replenish glutamate vesicles. Alternatively, glutamate is metabolized into α-KG that enters the TCA cycle and supplements ATP production. ATP is then used by Na^+^/K^+^ ATPases to re-establish the ionic gradient, replenishing the available ADP pool to stimulate glycolysis. Abbreviations: α-KG, α-ketoglutarate; ADP, adenosine diphosphate; ATP, adenosine triphosphate; EAAT, excitatory amino acid transporter; GCPR, G-protein-coupled receptor; Glc, glucose; Gln, glutamine; GLS, glutaminase; Glu, glutamate; GLUT, glucose transporter; GS, glutamate synthase; Lac, lactate; LDH, lactate dehydrogenase; MCT, monocarboxylate transporter; mGLUR, metabotropic glutamate receptor; NAD, nicotinamide adenine dinucleotide; NADH, nicotinamide adenine dinucleotide, reduced; OXPHOS, oxidative phosphorylation; PDH, pyruvate dehydrogenase; PKM1/M2, pyruvate kinase isozyme M1/M2; Pyr, pyruvate; TCA, tricarboxylic acid.

The brain is among the most metabolically active organs and it relies almost exclusively on glucose as its energy source (Howarth et al., 2012; Magistretti and Allaman, 2015; Ashrafi and Ryan, 2017; Dienel, 2019). It accounts for about 20% of total body oxygen consumption and 25% of total glucose usage while comprising only around 2% of total body weight (Sokoloff, 1977; Mink et al., 1981). Brain function relies on the partnership between neurons, glia and vasculature to meet such large metabolic requirements (Figure 2). Astrocytes are the most abundant and most relevant glia cells for brain energy metabolism (Barros et al., 2018; Jha and Morrison, 2018). They play several roles in maintaining proper brain function, some of which are directly linked to energy metabolism (Bélanger et al., 2011; Magistretti and Allaman, 2015). Astrocytes mediate the link between neuronal energy needs and circulating glucose by directly contacting and surrounding blood vessels with projections (endfeet), as well as regulating blood supply relative to neuronal demand (Oberheim et al., 2006; Gordon et al., 2008) by contributing to vasomotor responses (Attwell et al., 2010).

Astrocytes act as the main entry point of glucose into the brain via glucose transporters (GLUTs) expressed at both endothelium and astrocyte endfeet (Figure 2). This functional partnership establishes a crucial neurometabolic coupling that is the cornerstone of brain energy metabolism, enabling glia to sense neuronal energy needs and adjust the supply accordingly (Iadecola and Nedergaard, 2007; Mathiisen et al., 2010).

While both neurons and glia contribute toward brain energy metabolism, neuronal demands outweigh glia’s by far (Attwell and Laughlin, 2001; Alle et al., 2009; Nortley and Attwell, 2017). The main energy expenditure of neurons is associated with re-establishing membrane potentials and enabling synaptic transmission (Alle et al., 2009; Harris et al., 2012; Rangaraju et al., 2014). Thereby the activity of ion pumps such as the Na^+^/K^+^ ATPase accounts for the majority of total energy spent in the entire brain (Attwell and Laughlin, 2001; Harris et al., 2012). This holds true not only for re-establishing membrane ion gradients after membrane depolarization, but also for maintaining baseline membrane potential by compensating spike-related ion shifts (Attwell and Laughlin, 2001). It has been estimated that maintaining baseline membrane potential is responsible for close to half of the total energy consumption in the white matter (Harris and Attwell, 2012) and about a quarter in cortical neurons (Lennie, 2003). Surprisingly, membrane depolarization by itself does not require large amounts of ATP, as action potentials (AP) can persist for some time without ATP utilization (Rangaraju et al., 2014). On the other hand, Na^+^/K^+^ ATPase activity to restore the proper Na^+^ and K^+^ gradients after an AP consumes an immense amount of ATP (up to 8 × 10^8 molecules) (Lennie, 2003; Kole et al., 2008; Hallermann et al., 2012; Howarth et al., 2012). Interestingly, in synaptic transmission Na^+^/K^+^ ATPase activity represents only a small fraction of energy expenditure compared to the requirements of the synaptic vesicle cycle (Alle et al., 2009; Rangaraju et al., 2014; Yuan et al., 2018).

While neurons are primarily oxidative in their metabolism, relying on OXPHOS activity to generate ATP in an activity-dependent manner (Verstreken et al., 2005; Ly and Verstreken, 2006; Lin et al., 2010; Hall et al., 2012), astrocytes are highly glycolytic (Herrero-Mendez et al., 2009; Bittner et al., 2011; Figure 2). Several factors determine astrocytes’ metabolic profile: First, astrocytes have an increased expression and activity of Phosphofructokinase (PFK), thus promoting glycolysis (Herrero-Mendez et al., 2009; Bélanger et al., 2011; Zhang et al., 2014). Second, astrocytes express lactate dehydrogenase 5 (LDH5), an enzyme whose subunit composition favors catalysis of pyruvate into lactate (O’Brien et al., 2007; Zhang et al., 2014; Duka et al., 2017; Mason, 2017). Finally, astrocytes have reduced pyruvate dehydrogenase (PDH) activity, which means that any increase from basal pyruvate levels diverts into LDH5-mediated conversion to lactate instead of entry into TCA cycle and OXPHOS activity (Halim et al., 2010; Hirrlinger and Dringen, 2010; Zhang et al., 2014; Figure 2).

Conversely, neuronal glycolysis levels are negatively impacted by decreased synthesis and activity of PFK (Herrero-Mendez et al., 2009), an inability of neuronal pyruvate kinase (PKM1) to regulate glycolytic flux and finally, by expressing LDH1, a LDH isozyme whose subunit composition kinetically favors catalysis of lactate to pyruvate (Ward and Thompson, 2012; Zhang et al., 2014). The attenuation of glycolysis in neurons allows glucose to be diverted to the PPP in order to offset ROS production. Accordingly, upregulating glycolysis in neurons is met with increased oxidative stress and cell death (Gavillet et al., 2008; Herrero-Mendez et al., 2009; Bolaños et al., 2010).

The difference between neurons and astrocytes briefly outlined here is at the basis of their partnership and significantly shapes brain energy metabolism. This interdependence is formulated in the astrocyte-neuron lactate shuttle hypothesis (ANLS), one of the most widely accepted models describing brain energy metabolism at the cellular level (Pellerin and Magistretti, 1994; Pellerin et al., 1998; Allaman et al., 2011; Magistretti and Allaman, 2015). Underlying this model is the notion that even though glucose is the sole energy source of the brain, glucose-derived metabolites such as lactate can themselves serve as an alternate substrate for energy production.

The main postulate of the ANLS is that neuronal activation triggers an increase of astrocyte glucose uptake, which is then mainly converted into lactate and shuttled to neurons where it can be used as a substrate for oxidative energy metabolism (Pellerin and Magistretti, 1994; Magistretti and Allaman, 2015; Figure 2). In the ANLS seminal study, Pellerin and Magistretti demonstrated that glucose uptake and consequent lactate production in cultured astrocytes is stimulated by the excitatory neurotransmitter glutamate (Pellerin and Magistretti, 1994).

Glutamate released into the synaptic cleft is taken up by excitatory amino acid transporters (EAATs), which are present in glial lamellar processes sheeting the post- and pre-synaptic elements (Iadecola and Nedergaard, 2007). The extent of glutamate import serves as a neuronal activity measure and glutamate uptake by EAATs leads to a concomitant influx of Na^+^ and efflux of K^+^. This triggers Na^+^/K^+^ ATPase activity thereby decreasing local ATP concentration, which in turn promotes glycolysis in astrocytes (Barros et al., 2009; Herrero-Mendez et al., 2009; Chuquet et al., 2010; Bittner et al., 2011; Figure 2). Like neurons, astrocytes differentially express a pyruvate kinase isoform (PKM2) that, in contrast to the neuronal isoform, is capable of regulating glycolytic flux in response to increased energy needs (Zhang et al., 2014; Casson et al., 2016).

Astrocytic glycolysis feeds into lactate production which is released through dedicated monocarboxylate transporters (MCTs) (Pellerin et al., 2005). Neurons then take up extracellular lactate via neuron specific MCT where it is subsequently converted it into pyruvate by LDH1. Pyruvate then enters the TCA cycle, ultimately producing between 14 and 17 molecules of ATP through OXPHOS per molecule of lactate (Pellerin and Magistretti, 1994; Pellerin et al., 2005; Magistretti, 2006; Halestrap, 2013; Figure 2).

The large majority of the initial work behind the ANLS and in the years following its inception is based on *in vitro* cell models of neurons and astrocytes. Controversies concerning the ANLS mostly surrounded the question how readily these *in vitro* results are applicable to the situation in the intact brain (Tang, 2018; Dienel, 2019). However, as current research starts to focus on tissue culture and *in vivo* models, most of these questions are getting answered (Barros et al., 2009; van Hall et al., 2009; Chuquet et al., 2010; Wyss et al., 2011; Fünfschilling et al., 2012; Jakoby et al., 2014; Sotelo-Hitschfeld et al., 2015; Volkenhoff et al., 2015; Mongeon et al., 2016; Mächler et al., 2016; Clasadonte et al., 2017; Mazuel et al., 2017; Supplie et al., 2017; Zimmer et al., 2017; Ruminot et al., 2019). While the ANLS has been proposed to explain the metabolic landscape for the whole central nervous system (CNS), the canonical ANLS model cannot fully describe the observed metabolic interactions in the retina, despite being a part of the CNS. In the following section, we will consolidate and conceptually frame what is known about the retinal energy metabolism.

## Retinal Structure and Function

**FIGURE 3:**
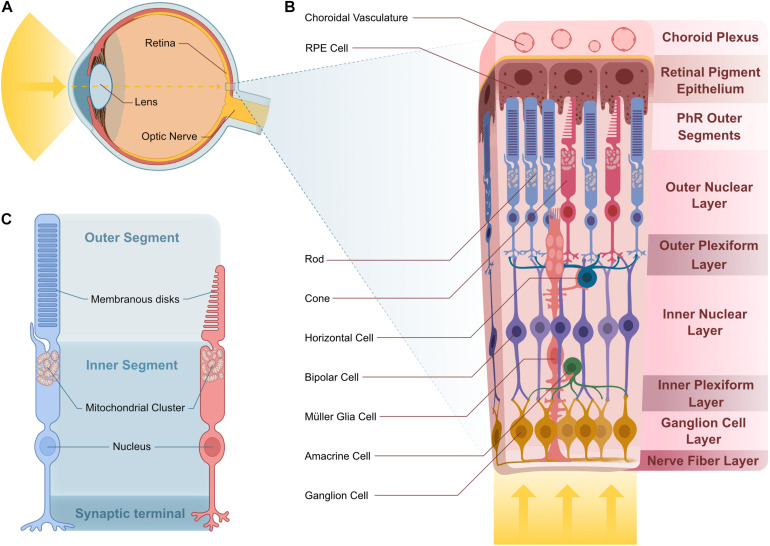
Vertebrate retina structure and cellular composition. **(A)** Sagittal representation of the human eye. Light enters the eye through the lens and is projected onto the retina. **(B)** Stereotypical multilayered structure of the canonical vertebrate retina. Light transverses the entire retina until it reaches the photosensible PhRs at the distal ONL, in contact with RPE projections that are adjacent to the choroid plexus where choroidal vasculature supplies nutrients to the outer retina. In the OPL, PhR terminals contact projections from HC and BC, whose cell body reside in the INL. Projections from BC contact those from the GC and AC in the IPL. GC cell bodies reside in the GCL and their projections bundle in the NFL to create the optic nerve. MGCs span all of these layers and support the cells they contact. **(C)** Rod and cone PhRs cell structure. PhRs have an inner and outer segment. PhR OSs house membranous disks that house the photosensitive pigment responsible for light detection. The IS consists of the rest of the cell body and houses a mitochondrial cluster at the base of the OS. Abbreviations: AC, amacrine cell; BC, bipolar cell; GC, ganglion cell; HC, horizontal cell; INL, inner nuclear layer; IPL, inner plexiform layer; IS, inner segment; MGC, Müller glia cell; NFL, nerve fiber layer; ONL, outer nuclear layer; OPL, outer plexiform layer; OS, outer segment; PhR, photoreceptor; RPE, retinal pigment epithelium.

In humans, vision is the primary sense and a large portion of the neocortex is dedicated to the processing of visual information. The first step in visual perception is light detection and pre-processing by the neural retina (Figure 3A). This stereotypically layered tissue has three nuclear layers (ONL, INL, GCL) and two synaptic layers (OPL and IPL) containing a total of five different neuronal and one glial cell type (Figure 3B). The ONL contains rod and cone photoreceptors, whereas cell bodies of bipolar, amacrine and horizontal cells, as well as Müller glia make up the INL. The third layer is the GCL, which contains ganglion cells as well as displaced amacrine cells. All of these cells contribute toward modulating and processing signals generated by photoreceptors in response to light (Dowling, 1970; Baden et al., 2020; Figure 3B).

Photoreceptors are the light-sensitive neurons, which are responsible for converting light into a biological signal in a process called phototransduction. Both rod and cone photoreceptors have an outer segment (OS) that contains membranous disks stacked with light-sensitive photopigments (Figure 3C). The photoreceptor outer segment is in direct contact with the retinal pigment epithelium (RPE), involved in numerous support functions and the apical part of the RPE is in contact with the choroidal vasculature (Bok, 1993; Figure 3B). This connection represents the photoreceptors’ main source for essential nutrients, establishing a major metabolite supply-line for photoreceptors as part of the Blood-Retina Barrier (BRB) (Campbell and Humphries, 2012).

## Retinal Energy Metabolism

The particularities of photoreceptor form and function are at the root of their high energy requirements (Wong-Riley, 2010; Country, 2017). Otto Warburg was the first to report on the levels of oxygen consumption of the retina, which was the highest of any tissue of the body that he tested, comparable only to rapidly proliferating cancer cells (Warburg et al., 1924; Warburg, 1956). Warburg first described the eponymous Warburg effect as a metabolic switch to glycolysis in the presence of oxygen (aerobic glycolysis) displayed by tumor cells. In addition to cancer cells, the Warburg effect is also found in the retina (Ng et al., 2015; DeBerardinis and Chandel, 2020). This is surprising, since in contrast to cancer cells, retinal neurons are postmitotic.

Apart from the high catabolic needs of photoreceptors, they display extraordinary anabolic needs. This is due to the curious phenomenon of outer segment renewal, where photoreceptors are continuously shedding the outer most part of the outer segments, amounting to about 10% per day (Young, 1967, 1971). Consequently, new disks have to be continuously added at the base of the outer segment, requiring a steady supply of a large number of complex macromolecules such as lipids and proteins to maintain proper function and OS length (Wang et al., 2011, 2016; Park et al., 2018; Léveillard et al., 2019).

**FIGURE 4:**
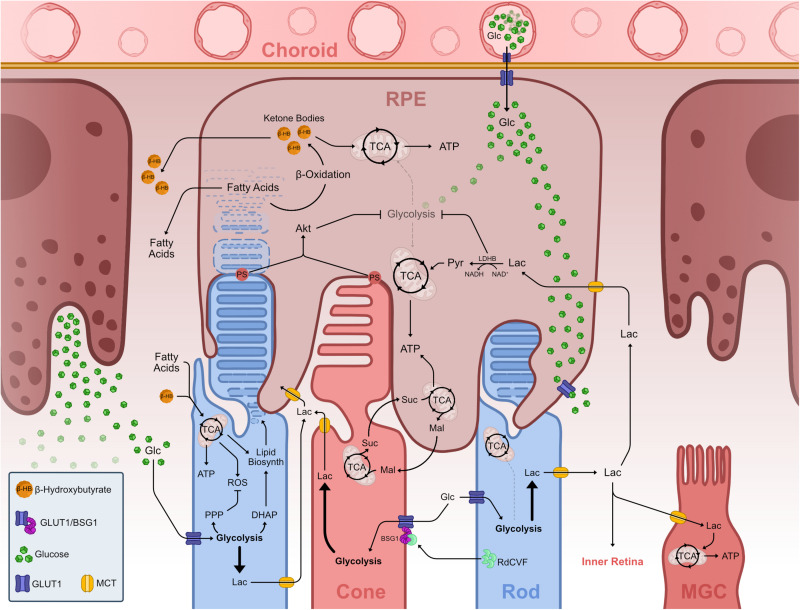
Metabolic landscape of the vertebrate outer retina. Representation of known metabolic interactions of cells within the outer retina. Glucose from the choroidal blood supply transverses the endothelium and enters the RPE via GLUT1. In the RPE, glucose flows through the cell un-metabolized, thus reaching the basal membrane adjacent to PhR OS. Glucose leaves the RPE via GLUT1 into the PhR layer. PhRs take up glucose via GLUT1 and use it to fuel aerobic glycolysis. Aerobic glycolysis in PhRs fuels the PPP, creating nucleic acids and offsetting ROS production from mitochondrial activity, and creates DHAP which is used to synthesize phospholipids necessary to create OS disks thus driving OS growth and PhR function. Lactate from aerobic glycolysis is transported from PhRs to the extracellular space where it is taken up by other cells such as MGC and RPE, fueling TCA cycle and OXPHOS in these cells. RPE cells take up lactate and transform into pyruvate, which enters the TCA. This LDHB activity depletes available NAD^+^, effectively inhibiting glycolysis, allowing glucose to reach GLUTs in the basal RPE membrane. Extending OS tips externalize PS and contact with RPE projections and subsequent phagocytosis leads to Akt activation, which also downregulates glycolysis in these cells. PhR OS phagocytosis in the RPE leads to an increase in available fatty acids from breaking down the membranous lipid disks. These fatty acids undergo β-oxidation creating ketone bodies in the form of β-hydroxybutyrate, which can be further metabolized and used in the TCA cycle, supplementing RPE mitochondrial activity. Both fatty acids and ketone bodies are transported to the PhR layer where they are taken up by these and used to supplement TCA cycle and OXPHOS. A reciprocal malate-succinate shuttle between PhRs and RPE cells exists that exchanges reducing power between these cells. In PhR, reverse SDH activity, due to hypoxia-induced decrease of COXIV, produces succinate that is exported to RPE cells, where it is oxidized in the TCA cycle and generates a surplus of malate. Malate is then exported to the PhR layer, enters the TCA cycle and fuels succinate production. Rods effectively promote cone survival and function by secreting RdCVF, which binds and makes a complex with BSG1 and GLUT1 in the cone membrane, promoting glucose supply to these cells. Abbreviations: ADP, adenosine diphosphate; Akt, protein kinase B; ATP, adenosine triphosphate; BSG1, basigin 1; COXIV, cytochrome c oxidase subunit 4; DHAP, dihydroxyacetone phosphate; Glc, glucose; GLUT, glucose transporter; Lac, lactate; LDH, lactate dehydrogenase; Mal, malate; MCT, monocarboxylate transporter; MGC, Müller glia cell; NAD, nicotinamide adenine dinucleotide; NADH, nicotinamide adenine dinucleotide, reduced; OS, outer segment; OXPHOS, oxidative phosphorylation; PDH, pyruvate dehydrogenase; PhR, photoreceptor; PPP, pentose phosphate pathway; PS, phosphatidylserine; Pyr, pyruvate; RdCVF, rod-derived cone viability factor; ROS, reactive oxygen species; RPE, retinal pigment epithelium; SDH, succinate dehydrogenase; Suc, succinate; TCA, tricarboxylic acid.

The retina, much like the rest of the CNS, preferentially uses glucose as its main energy substrate. Glucose supply of the retina is mainly mediated by glucose transporters (Hsu and Molday, 1991; Gospe et al., 2010), which are expressed in vasculature, RPE cells, PhRs and in MGCs (Watanabe et al., 1996; Wong-Riley, 2010; Veys et al., 2020; Figure 4). GLUTs are facilitative transporters and as such glucose follows a concentration gradient (Cheeseman and Long, 2015; Holman, 2020). Glucose from the blood supply leaves the choroidal endothelium and diffuses through RPE cells into the photoreceptor layer where photoreceptors take it up and readily metabolize it. This facilitated diffusion is thought to be mediated by GLUT1 (Wong-Riley, 2010; Narayan et al., 2017).

Like other neurons, the largest fraction of ATP expenditure within photoreceptors is active ion transport and membrane potential repolarization. ATP consumption in photoreceptors is activity dependent and substantially different in darkness and in light (Okawa et al., 2008; Hurley et al., 2015). The glutamate dependent dark current is energetically expensive and requires a large amount of ATP to sustain. In mice, a single rod photoreceptor consumes about four times more ATP in darkness than in light (approximately 10^8^ ATP s^–1^) (Okawa et al., 2008). Excess Ca^2+^ and Na^+^ is actively transported out of the cell via ATPase pumps and carries a significant energy cost. The combined cost for this efflux is almost 9 × 10^7^ ATP s^–1^. Interestingly, energy profiles for rods and cones differ quite substantially. In general, cones have higher energy needs than rods, probably due to differences in energy requirement of the dark current, phototransduction, and synaptic release (Okawa et al., 2008; Ingram et al., 2020).

As in most cells, a pivotal role of glucose in the retina is enabling ATP production to meet the cell’s energy demands (Ames et al., 1992; Ng et al., 2015; Narayan et al., 2017). Both glycolysis and OXPHOS generate ATP, although OXPHOS yields are far higher. It is by now well established that photoreceptors produce lactate in the presence of oxygen (Hurley et al., 2015; Kanow et al., 2017). As previously mentioned, aerobic glycolysis is also frequently referred to as the Warburg effect, which is a frequent trait of fast dividing cancer cells and neural stem cells (Namba et al., 2020). Why would energy-demanding cells like photoreceptors opt out of using OXPHOS, the most energy profitable pathway? At least a partial explanation may lie in the strong need for anabolic intermediates, provided by glycolysis (Casson et al., 2016; Narayan et al., 2017). Although photoreceptors do not proliferate, they nevertheless have an immense anabolic needs due to the constant renewal of their outer segments (Young, 1967, 1971; Ng et al., 2015).

Although the fascination with retinal energy metabolism has an almost century-long history starting with Warburg, more recent work has now laid the foundation for a more integrative view of retinal energy metabolism. The focus is no longer solely on photoreceptors but on the metabolic network of neighboring cells in the outer retina forming a metabolic landscape (Figure 4). The first consideration is how glucose enters the retina. Glucose is provided by blood circulation and has to pass the blood retina barrier (BRB). It is shuttled through the choroidal endothelium into adjacent RPE cells, before reaching the outer retina. RPE shuttles most glucose via GLUT1 to the photoreceptor layer, while relying on lactate provided by photoreceptors for its own metabolism (Badr et al., 2000; Gospe et al., 2010; Kanow et al., 2017; Figure 4). RPE cells minimize glucose consumption and maximize glucose efflux by using photoreceptor-derived lactate as their metabolic substrate. Concomitantly, lactate is a negative feedback regulator of glycolysis that dampens RPE glucose consumption (Figure 4; Kanow et al., 2017). This intimate metabolic co-dependence is not restricted to catabolism, but extends to anabolism. RPE cells export malate for photoreceptors to replenish succinate that is exported back to RPE cells. This enables RPE cells to generate reductive power in the form of NADPH regenerating malate in the process, thereby closing the cycle (Bisbach et al., 2020; Figure 4). Malate can also be converted to pyruvate, yielding additional NADPH in the process (Adler et al., 2014; Figures 1, 4). RPE cells are also known to rely on β-oxidation of fatty acids, resulting from phagocytosed disks of shed photoreceptor OS, to generate energy. The resulting ketone bodies supplement the TCA cycle, providing ATP and metabolic intermediates, and therefore compensate for the downregulation of glycolysis (Figure 4; Adijanto et al., 2014; Reyes-Reveles et al., 2017; Fu et al., 2020). Moreover, the RPE has been shown to supply photoreceptors with fatty acids and ketone bodies, which can be metabolized in the mitochondria and supplement TCA cycle activity and ATP production (Figure 4; Adijanto et al., 2014; Léveillard and Sahel, 2017; Fu et al., 2020). Another hallmark of the interdependence between photoreceptor outer segments and the RPE is the recent finding that contacts between phosphatidylserine on outer segment tips and RPE receptors activates the intracellular signaling Akt pathway. This effectively links phagocytosis with glucose transport to photoreceptors for new outer segment synthesis (Wang et al., 2019; Figure 4).

Taken together, these studies establish a close metabolic interaction between the RPE and the outer retina regarding the exchange of not only glucose and lactate, but also metabolites involved in anabolism. The involvement of aerobic glycolysis in biogenesis and maintenance of outer segment has been established also in rod photoreceptors, by interfering with key enzymes mediating aerobic glycolysis (Chinchore et al., 2017; Petit et al., 2018).

In the metabolic landscape there are not only reciprocal interactions between the RPE and photoreceptors, but also between rods and cones. The interdependence of photoreceptors has been known for long by the puzzling bystander effect where genetically compromised rods lead to the demise of uncompromised cones (Ripps, 2002). This effect is proposed to be due to the absence of the rod-derived cone viability factor (RdCVF) resulting from rod degeneration. The splice variant of the Nxnl1genes encodes RdCVF, which binds to Basigin-1 (BSG1) expressed on the cell surface of cones. BSG1 forms a complex with GLUT1, stimulating glucose uptake upon RdCVF binding and consequently aerobic glycolysis (Léveillard et al., 2004; Aït-Ali et al., 2015; Figure 4). The absence of this interaction metabolically starves cones.

In the proposed metabolic landscape scenario, photoreceptors are set as energy relay nodes using glucose to meet their anabolic and catabolic needs. At the same time, they provide surrounding cells with metabolic intermediates (Figure 4). This is a fascinating variation on the ANLS in other parts of the CNS. We saw how the ANLS depicts a metabolic exchange between astrocytes and neurons where astrocytic lactate from aerobic glycolysis helps fuel neuronal activity. Within the retinal metabolic landscape, photoreceptors take on a similar role to astrocytes in the rest of the CNS by supplying lactate to surrounding cells. Moreover, both astrocytes in the CNS and photoreceptors in the retina are the first neural cells to contact glucose in their respective system. This curious “role-reversal” may very well be an adaption to the unique microenvironment of the retina and the extraordinary anabolic needs of postmitotic photoreceptors. The large demand of glycolytic intermediates for anabolism makes photoreceptors a glucose sink that surrounding cells have adapted to by using by-products to support their own metabolic needs (Kanow et al., 2017).

This model suggests an intricate and interdependent cell environment, yet there are some interesting aspects that await further clarification. Among them is the metabolic distinction between rod and cone photoreceptors and use-dependent metabolic requirements. Another curious outstanding question is how photoreceptors cope with the low ATP-yielding aerobic glycolysis to support oxidative metabolism alone. Moreover, if this is the case then why do photoreceptors have large, tightly packed mitochondrial clusters adjacent to the OS (Giarmarco et al., 2020)? While the current retinal metabolic landscape specifies many interactions within the retina from a bioenergetics point-of-view, it is far from complete and many of the proposed interactions would benefit from additional robustness and independent confirmation. Furthermore, almost all of the supporting data is from murine models that rely mainly on scotopic (nocturnal) vision and whose retina is composed of only about 2% cones. The rest of the data is from *in vitro* cell modeling of the retina. While both these approaches have proven important, there is a pressing need for further *in vivo* data avoiding more artificial *in vitro* methods. This is particularly true for information on cone photoreceptors, which mediate high-acuity color vision that humans rely on.

## Retinal Metabolism in Disease

We have summarized recent evidence that glucose metabolism is the key to maintaining brain and retina homeostasis. Given the strong dependence of neural cells on glucose metabolism, it comes as no surprise that metabolism is frequently affected in neural diseases, be it the cause or the results of metabolic perturbations. The retina is no exception to this and several retinal diseases have been linked to metabolism.

A case in point are the pathomechanisms of outer retinal dystrophies, such as of age-related macular degeneration (AMD) and retinitis pigmentosa (RP). In this group of diseases, there is a growing realization that metabolic alterations may lie at the heart of their pathophysiology (Narayan et al., 2016; Campochiaro and Mir, 2018; Léveillard et al., 2019).

Age-related macular degeneration is a retinal disease that is the leading cause of blindness in seniors worldwide (Mitchell et al., 2018). While the mechanisms behind AMD have yet to be uncovered, several recent studies point to oxidative damage (Ferrington et al., 2017; Hwang et al., 2019; Terluk et al., 2019) and mitochondrial dysfunction as key steps in AMD pathophysiology (Karunadharma et al., 2010; Terluk et al., 2015; Ebeling et al., 2020). The concept of a retinal metabolic landscape can help to link accumulated mitochondrial DNA damage in the RPE to macular degeneration. RPE cells with compromised mitochondrial function may shift toward increased glucose consumption and thereby compromise photoreceptor access to glucose. Conversely, changes in photoreceptor energy supply will have repercussions on the RPE.

Retinitis pigmentosa (RP) is the disease most tightly linked to changes in the retinal metabolic landscape. The retinal metabolic landscape brought forward here was strongly driven by studies related to RP (Aït-Ali et al., 2015; Wang et al., 2016, 2019; Petit et al., 2018). RP describes a group of rare diseases where mostly rod-specific mutations cause rod, and subsequently cone, degeneration (Hartong et al., 2006). The reason behind this bystander effect can be attributed to the lack of cone viability factor (RdCVF), which ultimately stimulates cone glucose uptake (Aït-Ali et al., 2015).

As the intricacies of the retinal metabolic landscape start to unravel, we begin to see how energy homeostasis is crucial to maintain a proper functioning system. Upsetting metabolic interactions can have significant consequences leading to specific disease phenotypes. Furthering our knowledge of this fascinating retinal landscape on any level is bound to have a large impact on what is already known and has yet to be discovered about these diseases, as well as possibly contributing to the development of therapeutic strategies to combat them.

## Author Contributions

FV and SN drafted, wrote, and edited the manuscript. FV designed and drew the figures. Both authors contributed to the article and approved the submitted version.

## Conflict of Interest

The authors declare that the research was conducted in the absence of any commercial or financial relationships that could be construed as a potential conflict of interest.

## Abbreviations

ADP: adenosine diphosphate
AMD: age-related macular degeneration
ANLS: astrocyte-neuron lactate shuttle
AP: action potential
ATP: adenosine triphosphate
BRB: blood-retina barrier
BSG: basigin
CNS: central nervous system
DHAP: dihydroxyacetone phosphate
EAAT: excitatory amino acid transporter
FAD: flavin adenine dinucleotide
FADH: flavin adenine dinucleotide hydroquinone
G6P: glucose-6-phosphate
GCL: ganglion cell layer
GLUT: glucose transporter
HK: hexokinase
INL: inner nuclear layer
IPL: inner plexiform layer
LDH: lactate dehydrogenase
MCT: monocarboxylate transporter
MGC: müller glia cell
NAD: nicotinamide adenine dinucleotide
NADH: nicotinamide adenine dinucleotide hydrogen
NADP: nicotinamide adenine dinucleotide phosphate
NADPH: nicotinamide adenine dinucleotide phosphate hydrogen
ONL: outer nuclear layer
OPL: outer plexiform layer
OS: outer segment
OXPHOS: oxidative phosphorylation
PDH: pyruvate dehydrogenase
PFK: phosphofructokinase
PhR: photoreceptor
PKM1/M2: pyruvate kinase isozyme M1/M2
PPP: pentose phosphate pathway
RdCVF: rod-derived cone viability factor
ROS: reactive oxygen species
RP: retinitis pigmentosa
RPE: retinal pigment epithelium
TCA: tricarboxylic acid.
